# Lack of cardiac benefit after intramyocardial or intravenous injection of mesenchymal stem cell-derived extracellular vesicles supports the need for optimized cardiac delivery

**DOI:** 10.20517/2574-1209.2023.98

**Published:** 2023-12-21

**Authors:** Cynthia M. Xu, Mark Broadwin, Patrick Faherty, Rayane Brinck Teixeira, Mohamed Sabra, Frank W. Sellke, M. Ruhul Abid

**Affiliations:** 1 Cardiovascular Research Center, Rhode Island Hospital, Providence, RI 02903, USA.; 2 Division of Cardiothoracic Surgery Alpert Medical School of Brown University and Rhode Island Hospital Providence, Providence, RI 02903, USA.

**Keywords:** Human bone mesenchymal stem cell-derived extracellular vesicles, biodistribution, intramyocardial injection, intravenous injection, myocardial infarction

## Abstract

**Aim:**

To determine the differences in improvement in cardiac function by intramyocardial (IM) *vs*. intravenous (IV) injection of human bone mesenchymal stem cell-derived extracellular vesicles (HBMSC-EV) after acute MI.

**Methods:**

FVB mice underwent acute MI via left anterior descending coronary artery ligation and subsequent injection of: (1) IM saline control; (2) IM HBMSC-EV; (3) IV saline control; and (4) IV HBMSC-EV. Cardiac function was evaluated with weekly postoperative echocardiography. On postoperative day 28, the mice were euthanized, and the heart, lungs, liver, spleen, and kidneys were harvested. Given previous studies showing HBMSC-EV hepatic uptake after IV injection, the liver was evaluated for changes in inflammation, fibrosis, and proliferation.

**Results:**

On postoperative day 28, there were no significant differences in left ventricular ejection fraction (*P* = 0.6151), fractional shortening (*P* = 0.1135), or anterior border zone fibrosis (*P* = 0.6333) in any of the experimental groups. Interestingly, there was a strong trend demonstrating improvement in infarct size on fibrosis staining, which did not reach significance (*P* = 0.05620). There were no differences in hepatic inflammation, fibrosis, and proliferation.

**Conclusions:**

Although there was a trend in the improvement in infarct size, a single-dose administration of neither IM nor IV injection of HBMSC-EV resulted in significant improvement in post-MI cardiac function. A major limitation of this study is the lack of trials determining the optimal dose of HBMSC-EV needed in this model. However, the current study demonstrates that future studies are required to either optimize administration or bioengineer HBMSC-EV with cardiac-homing properties.

## INTRODUCTION

Extracellular vesicles (EV) are secreted by almost every cell type and are lipid bilayer membranes that encase a variety of compounds such as proteins, lipids, nucleic acids, *etc*. In stem cells, EVs are thought to be responsible for the beneficial paracrine effects. Stem cell-derived EVs have a wide range of therapeutic functions in preclinical models of cardiovascular disease - EVs have been shown to protect several forms of myocardial ischemia in both small and large animal models^[1–4]^.

Furthermore, intramyocardial (IM) injection has been shown to be the most reliable form of EV delivery, while intravenous (IV) injection is not^[1,2,4–7]^. A systemic review found that when EVs were intravenously injected, there was little to no evidence of EV localization to the heart, with primarily hepatic and splenic uptake^[8]^. We recently confirmed these findings in a model of murine acute MI, and only found cardiac uptake of EVs after IM injection and not with IV injection^[7]^.

However, no long-term functional studies have been done to compare the effects of intramyocardial *vs*. IV EV injection side-by-side in an acute MI model. These two different delivery methods are important to compare since IM injection has more reliable cardiac benefits in preclinical studies, but requires a thoracotomy to deliver. IV injection, though with less reliable cardiac benefits, is a non-invasive delivery method that is more translatable to clinical practice. In this study, we delivered human bone mesenchymal stem cell-derived EVs (HBMSC-EV) via either IM or IV injection after murine acute MI and examined cardiac function 28 days post-injection. Additionally, as our previous findings confirmed hepatic HBMSC-EV uptake after IV injection, we evaluated for evidence of changes in liver inflammation, fibrosis, growth, and oxidative status 28 days post-injection since mesenchymal stem cell-derived EVs have been shown to have beneficial effects in preclinical liver disease models^[9]^. We also wanted to determine if there were any detectable long-term changes in the liver after HBMSC-EV uptake.

## METHODS

### HBMSC-EV isolation

HBMSC (Lonza, PT-2501) were cultured to passage 7 with the Mesenchymal Stem Cell Growth Medium (MSCGM) BulletKit (Lonza, PT-3001), per manufacturer’s instructions^[7]^. At 80%−90% confluency, the old MSCGM was removed from the HBMSC and replaced with fresh MSCGM. The HBMSC were placed in a humidified hypoxia chamber (Billups-Rothenberg, MIC-101) containing 95% N_2_ and 5% CO_2_ for 24 h at 37 °C. The HBMSC were then removed from the hypoxia chamber and the media was collected. The media was centrifuged at 2,000× *g* to remove the cell debris, and then ultracentrifuged (WX Ultra Centrifuge with Sorvall AH-629 rotor) twice at 100,000× *g* for 70 min to isolate the HBMSC-EV pellet and to wash the HBMSC-EV with Dulbecco’s Phosphate Buffered Saline (PBS). The HBMSC-EV were re-suspended in PBS with 1% dimethylsulfoxide (DMSO) and stored at −80 °C^[7]^. Hypoxia pre-conditioned HBMSC-EVs were isolated, given previous studies suggesting greater proteomic promise in cardiovascular disease^[10]^. Using this protocol, approximately 5–7 million HBMSC were needed to produce the injection dosage, 2 × 10^9^ HBMSC-EV particles.

### HBMSC-EV characterization

As previously described, the HBMSC-EV were characterized through electron microscopy, nanoparticle tracking analysis, and immunoblotting^[7]^. The HBMSC-EV were visualized with electron microscopy (FEI Morgagni 268) after fixation and contrasted in 4% uranyl acetate. The size, number, and distribution of the HBMSC-EV were quantified with the Nanosight NS500 (Malvern Instruments). 10 μg of HBMSC-EV protein was loaded in a Bis-Tris gel and run using MOPS-SDS running buffer. The following markers were evaluated through immunoblotting: CD81 (Cell Signaling #52892S, 1:1,000), CD9 (Cell Signaling #13403S, 1:1,000), Alix (Cell Signaling #92880S, 1:1,000), GAPDH (Cell Signaling #97166S, 1:1,000), heat shock protein 70 (HSP70) (Cell Signaling #4872T, 1:1,000), and albumin (Cell Signaling #4929S, 1:1,000)^[11]^.

### Animals

Female and male FVB/NCrl mice (8–10 weeks old, Charles River Stock No. 207) were acclimatized and housed at the Coro Building Barrier facility. Experiments were carried out in accordance with the approved protocol via the Institutional Animal Care and Use Committee (Protocol 1844667/CMTT# 5017–22).

### Echocardiogram

Echocardiogram (Vevo 2100, FUJIFILM VisualSonic Inc.) was performed preoperatively, and postoperatively on postoperative days 3, 7, 14, 21, and 28. The mice were anesthetized with 2% isoflurane and monitored to ensure normothermia and heart rate between 400–600 beats per minute. Left heart systolic function was evaluated by obtaining two-dimensional parasternal long-axis views, and proximal, mid-papillary and distal short-axis views. Through Simpson’s method on Vevo Lab 5.6.0, left ventricular ejection fraction (LVEF) and fractional shortening (FS) were quantified.

### Surgical procedure: left anterior descending (LAD) coronary artery ligation

Anesthesia was induced with 3% isoflurane and ketamine (100 mg/kg). Buprenorphine SR (1 mg/kg) was administered in the dorsal fat pad. The mice were intubated and ventilated (MiniVent Type 845, Harvard Apparatus) and maintained at 2% isoflurane. A left thoracotomy in the 3rd interspace was made to expose the heart. The LAD artery was ligated with an 8–0 nylon suture 2–3 mm below the left atrial appendage with resulting blanching and dyskinesia^[12]^.

**Figure 1.**
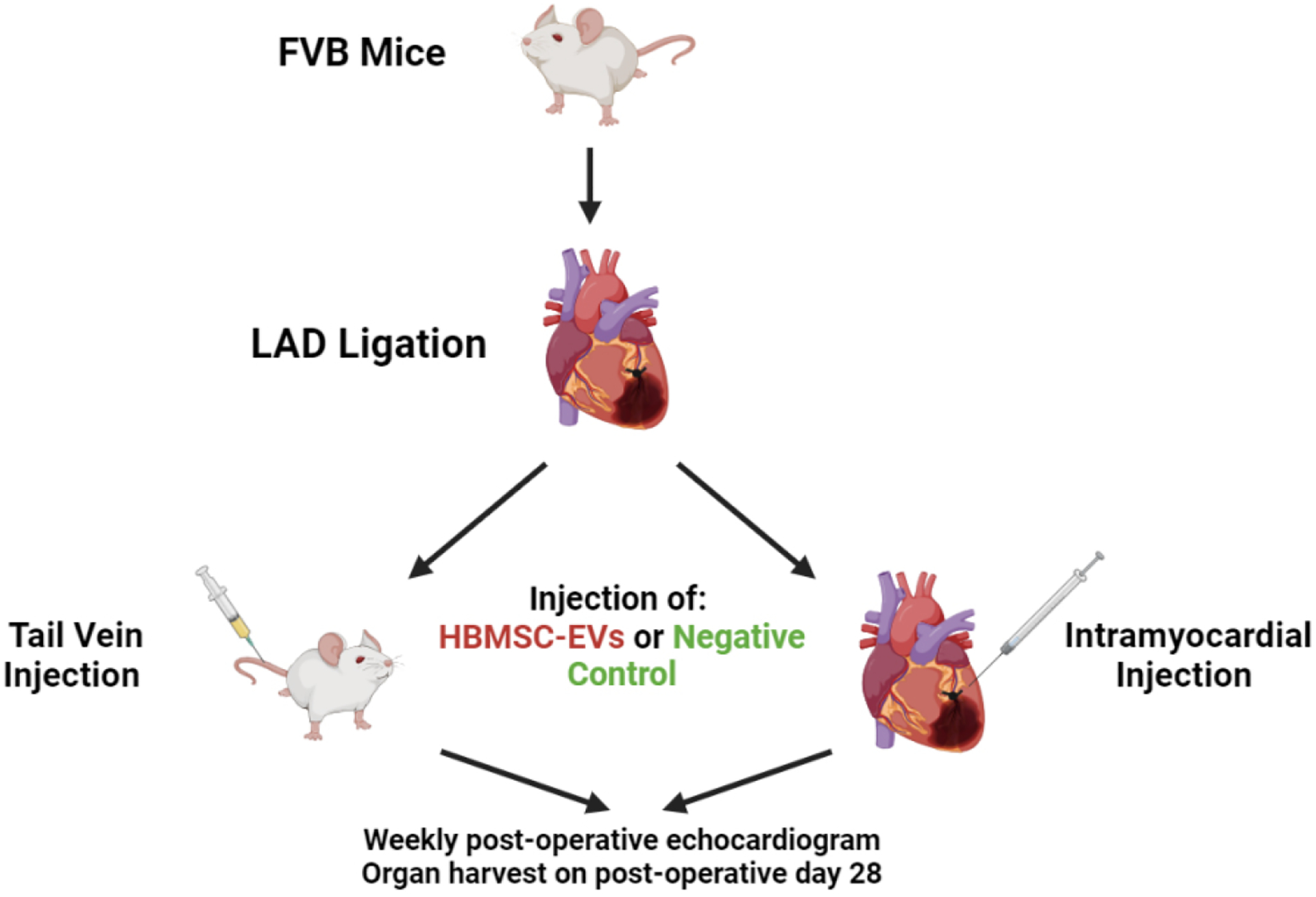
Wild-type mice underwent left anterior descending coronary artery (LAD) ligation to induce myocardial infarction, and then were split into a tail vein or intramyocardial injection group to receive either human bone mesenchymal stem cell-derived extracellular vesicles (HBMSC-EV) or a negative saline control. The mice were then allowed to recover postoperatively, and underwent postoperative echocardiogram to evaluate cardiac function. The mice were euthanized on postoperative day 28 for organ harvest. This image was created using BioRender.com and used with permission.

Immediately after ligation, the mice received one of the following injections [Figure 1]:

1. IM injection control (IM-C) (*n* = 8): 10 μL PBS with 1% DMSO.
2. IM injection HBMSC-EV (IM-EV) (*n* = 9): 2 × 10^9^ HBMSC-EV particles in 10 μL PBS with 1% DMSO.
3. Tail vein injection control (IV-C) (*n* = 8): 10 μL PBS with 1% DMSO + 190 μL PBS.
4. Tail vein injection HBMSC-EV (IV-EV) (*n* = 8): 2 × 10^9^ HBMSC-EV particles in 10 μL PBS with 1% DMSO + 190 μL PBS.

The dosage of 2 × 10^9^ HBMSC-EV particles was chosen based on previous biodistribution studies that visualized positive organ uptake at this dosage 2 h after injection^[7]^. The intramyocardial injection was done with a Neuros Syringe (Hamilton, 1183U32) in two locations (5 μL per location) - immediately below the stitch in the ischemic myocardium and 1–2 mm lower in the anterior ischemic myocardium after clear myocardial blanching was visualized. The tail vein injection was done with a 0.5 mL insulin syringe. The thoracotomy was closed with absorbable sutures and the pneumothorax was evacuated. The mice were awakened from anesthesia, extubated, and allowed to recover in a warm cage.

On postoperative day 28, the mice were euthanized and the heart, lungs, liver, spleen, and kidneys were harvested.

### Histology

The hearts and livers were embedded in Tissue-Tek OCT (Sakura Finetek) and were stored at −80 °C. Sectioning was done in 5 μm thickness. At the mid-papillary level, the heart was evaluated for infarct size and anterior border zone interstitial fibrosis with Masson-Trichrome staining. Quantification was done through ImageJ.

Given the previous positive hepatic uptake of HBMSC-EV after IV delivery, the liver tissues of the IV-C and IV-EV groups were further evaluated. To look for changes in liver inflammation, fibrosis and proliferation, hematoxylin and eosin, Masson-Trichrome and immunofluorescence staining of proliferating cell nuclear antigen (PCNA) (Cell Signaling #2586, 1:400) were performed, respectively. The hematoxylin and eosin images were examined for leukocyte infiltration. Fibrosis was quantified using ImageJ. Positive PCNA staining was determined via manual counting.

### Immunoblotting

In a Bis-Tris gel, 10 μg of protein from liver tissue lysate was loaded and run in MOPS-SDS running buffer. The following markers were evaluated through immunoblotting: tumor necrosis factor alpha (TNF-α) (Cell Signaling #11948, 1:1,000), transforming growth factor beta (TGFβ) (Cell Signaling #3711, 1:1,000), SMAD 2/3 (Cell Signaling #8685, 1:1,000), mammalian target of rapamycin (mTOR) (Cell Signaling #2983, 1:1,000), B-cell lymphoma 2 (Bcl-2) (Cell Signaling #3498, 1:1,000), superoxide dismutase 1 (SOD1) (Cell Signaling #37385, 1:1,000), superoxide dismutase 2 (SOD2) (Cell Signaling #13141, 1:1,000), phospho-endothelial nitric oxide synthase (p-eNOS, Ser1177) (Cell Signaling #9570, 1:1,000), endothelial nitric oxide synthase (eNOS) (Cell Signaling #32027, 1:1,000), and beta-actin (Cell Signaling #58169, 1:1,000). The following secondary antibodies were used: anti-mouse IgG, HRP-linked antibody (Cell Signaling #7076, 1:5,000), and anti-rabbit IgG, HRP-linked antibody (Cell Signaling #7074, 1:5,000). The band intensities were normalized by beta-actin and quantified on ImageJ.

### Statistical analysis

Continuous data were obtained from echocardiogram, histological, and immunoblotting results. Data analysis was performed on GraphPad Prism 9.0.2. The Shapiro-Wilk test was done to check for normality. For pairwise comparisons, either the unpaired *t*-test (parametric data) or the Mann-Whitney test (non-parametric data) was used. For comparisons of more than two groups, the one-way ANOVA (parametric) or Kruskal-Wallis (non-parametric) tests were used, followed by the post-hoc Tukey (parametric) or Dunn (non-parametric) tests.

## RESULTS

### No long-term improvements in cardiac recovery were found after HBMSC-EV injection, when delivered either IM or IV

**Figure 2.**
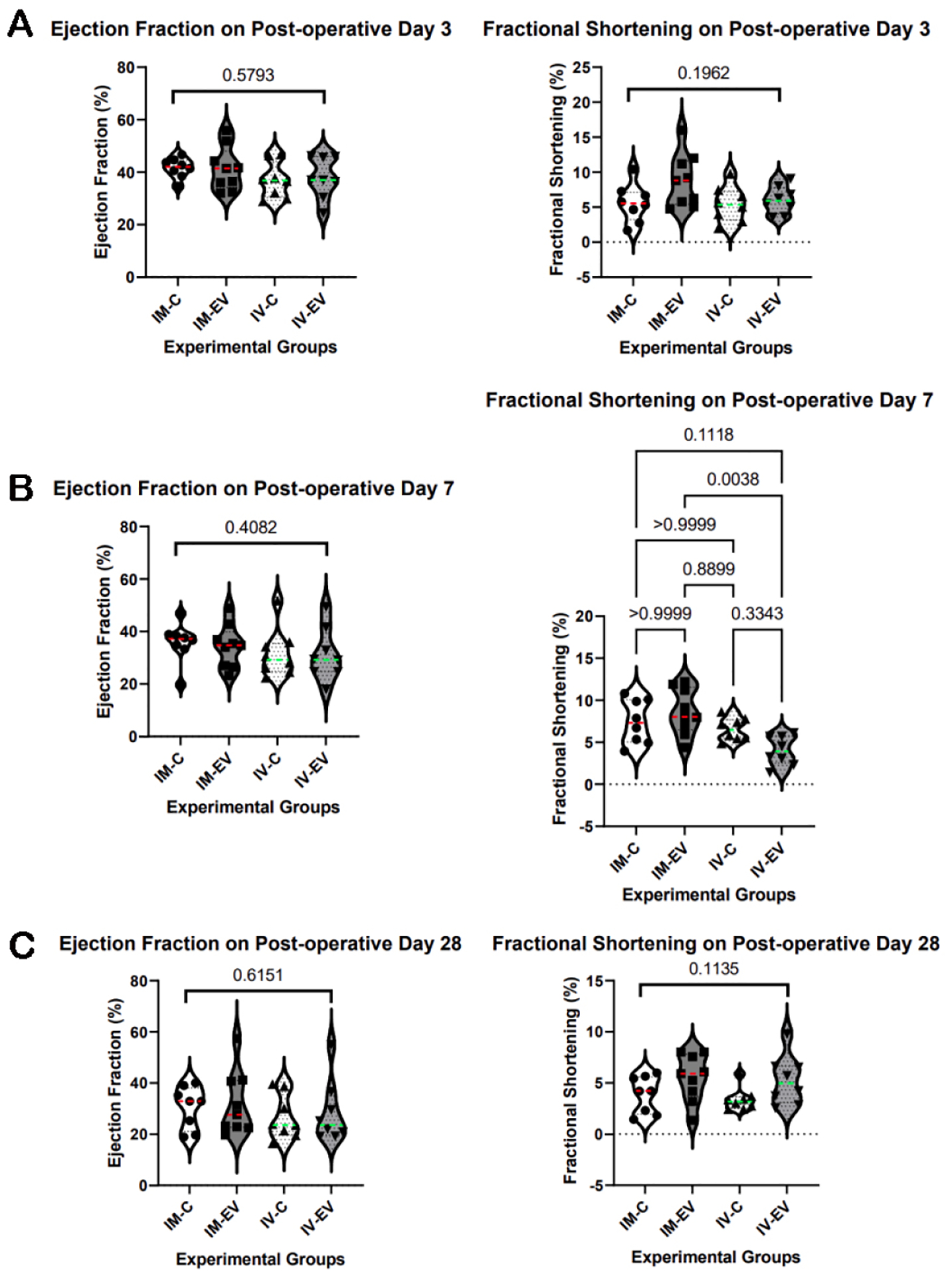
There were no significant long-term benefits in left ventricular systolic function. (A) There were no differences in left ventricular ejection fraction (LVEF) or fractional shortening (FS) on postoperative day 3. (B) There were no differences in LVEF on postoperative day 7, but the FS of IM-EV was significantly greater than that of IV-EV (*P* = 0.0038). (C) On postoperative day 28, there were no differences in either LVEF or FS; IM-C, IM control injection group (*n* = 8); IM-EV, IM extracellular vesicles injection group (*n* = 9); IV-C, IV control injection group (*n* = 8); IV-EV, IV extracellular vesicle injection group (*n* = 8). The Shapiro-Wilk test was used to determine that the data were non-parametric. Then, the Kruskal-Wallis test was performed, as well as the post-hoc Dunn’s test when appropriate. The median value of each group is displayed in color on the violin plot of each group.

Preoperatively, all mice had normal cardiac function on echocardiogram. Overall, there were no significant changes in either LVEF or FS by postoperative day 28 [Figure 2]. The FS of IM-EV was significantly greater than that of IV-EV on postoperative 7 (*P* = 0.0038), but these differences disappeared with time.

**Figure 3.**
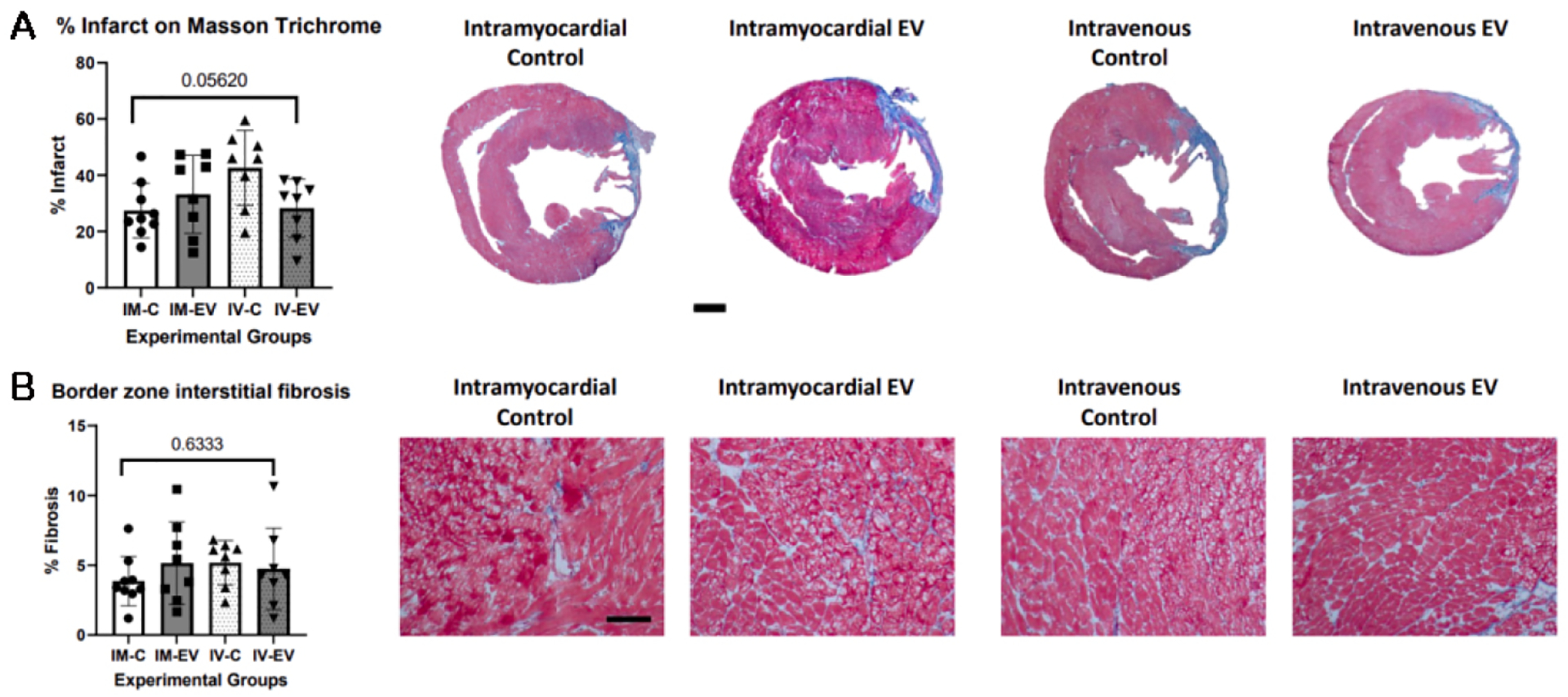
There were no significant changes in myocardial infarct size or border zone interstitial fibrosis after either IM or IV human bone mesenchymal stem cell-derived extracellular vesicle (HBMSC-EV) injection. (A) The infarct percentages of all four groups were not found to be significantly different (*P* = 0.05620). Representative images of the infarct size in each of the four groups are shown. Scale bar = 1 mm. (B) Anterior border zone interstitial fibrosis was not found to be significantly different (*P* = 0.6333). Scale bar = 100 μm; IM-C, IM control injection group; IM-EV, IM extracellular vesicle injection group; IV-C, IV control injection group; IV-EV, IV extracellular vesicle injection group. The Shapiro-Wilk test was used to determine that the data were parametric. Then, the one-way ANOVA test was performed.

Quantification of myocardial infarct after Masson-Trichrome staining showed that although there was a trend in differences in infarct size among the four groups, infarct size differences did not reach significance by IM or IV administration of HBMSC-EV (*P* = 0.05620) [Figure 3A]. There were also no significant changes in border zone interstitial fibrosis (*P* = 0.6333) [Figure 3B].

### Intravenously delivered HBMSC-EV do not affect hepatic inflammation, fibrosis or proliferation in the absence of liver disease

**Figure 4.**
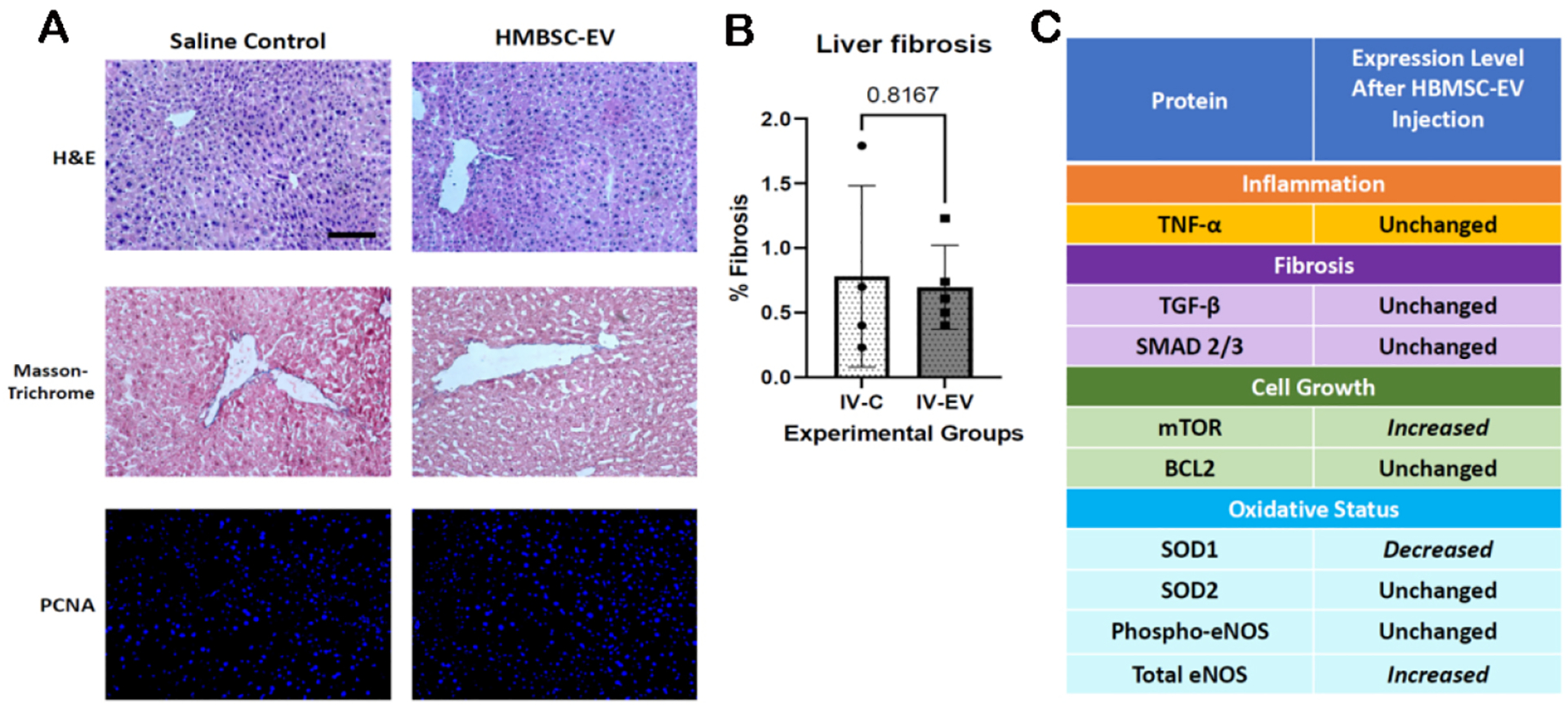
Intravenous (IV) injection of human bone mesenchymal stem cell-derived extracellular vesicles (HBMSC-EV) did not alter hepatic inflammatory status, fibrosis, or proliferation. (A) Hematoxylin and eosin staining, Masson-Trichrome staining, and proliferating cell nuclear antigen (PCNA) immunofluorescence did not demonstrate differences in leukocyte infiltration, fibrosis, and cellular proliferation, respectively. Scale bar = 100 μm. (B) Quantification of % fibrosis on Masson-Trichrome staining was not significantly different (*P* = 0.8167) between the IV control (IV-C) and IV HBMSC-EV (IV-EV) injection groups. Statistical analysis was performed using the Shapiro-Wilk and unpaired t-test. (C) Immunoblotting showed no differences in major regulators of inflammatory and fibrosis pathways, such as tumor necrosis factor-alpha (TNF-α), transforming growth factor-β (TGF-β), and SMAD 2/3. Cell proliferation regulator mammalian target of rapamycin (mTOR) expression was increased in the IV-EV group, but this finding is difficult to interpret given the complexity of mTOR regulation and the negative PCNA findings. Oxidative protein expression changes are difficult to interpret as well, given the lack of hepatic oxidative injury in this model.

Hepatic uptake of IV HBMSC-EV after murine MI was demonstrated in previous studies, but this current study did not demonstrate any long-term effects of the HBMSC-EV in the absence of liver injury [Figure 4A]^[7]^. Hematoxylin and eosin staining showed no leukocyte infiltrates upon review. Masson-Trichrome staining did not demonstrate differences in fibrosis (*P* = 0.8167) [Figure 4B]. PCNA immunofluorescence was negative for both control and HBMSC-EV groups, as expected in the absence of liver injury or malignancy.

Concurrent immunoblotting of major signaling proteins of inflammatory and fibrosis and proliferative pathways supported the histological results [Figure 4C]. Expression of tumor necrosis factor-alpha (TNF-α), a major regulator of the inflammatory process, and some of the key fibrotic pathway markers transforming growth factor-β (TGF-β) and SMAD 2/3 was not changed after IV HBMSC-EV (*P* = 0.3944, *P* = 0.1961, respectively). The mammalian target of rapamycin (mTOR) is involved with cellular growth and had increased expression (*P* = 0.0066) after HBMSC IV injection, but given the complexity of its regulation, this finding is difficult to interpret. B-cell lymphoma 2 (Bcl-2), an apoptosis regulator, was not significantly different (*P* = 0.8055). Proteins involved in oxidative stress were evaluated as well, and although expression of superoxide dismutase 1 (SOD1) and total endothelial nitric oxide synthase (eNOS) were altered after HBMSC-EV injection (*P* < 0.0001, *P* = 0.0334, respectively), these findings are difficult to interpret since there was no hepatic oxidative injury in this model.

## DISCUSSION

The findings of this study clearly show that a single dose of either IM or IV HBMSC-EV injection does not improve cardiac outcomes significantly after acute MI. It is intriguing that although previous studies have shown cardiac benefits with IM injection (2, 4, 5, 13), the current study did not. There may be several reasons for our findings in the present study. Acute MI represents a severe form of cardiac injury where our single dose injection of HBMSC-EV may not be sufficient to achieve a meaningful effect. Our dose was 2 × 10^9^ HBMSC-EV particles isolated from wild-type HBMSC with no modifications. It is unclear if this dose can be considered sufficient since one of the main limitations in reviewing EV research is the lack of dosage standardization or quantification in either μg EV protein or EV particles. Examples of dosages that have been used with cardiac improvements range from 50 μg protein in porcine models in chronic myocardial ischemia, 33 × 10^11^ particles in myocardial ischemia-reperfusion and convalescent MI in porcine models, to 200 μg protein in a murine acute MI model^[4,6,13]^. Additionally, EV cell sources are widely varied, even among EVs isolated from similar cell types, given the likely differences in donors. However, given the large number of HBMSC that we needed to isolate 2 × 10^9^ HBMSC-EV particles for this small animal model, we do not think that our dose should be considered insufficient. Furthermore, optimizing HBMSC-EV administration timing is difficult to do in a murine model, as re-do thoracotomy and intramyocardial injection is prohibitive due to operative scarring from the initial procedure. In a large animal model, re-do thoracotomy is possible but is far more expensive. If HBMSC-EV were able to be effectively delivered to the heart non-invasively, there would be far more flexibility in testing dosing timing. Perhaps also a cardiac benefit might have been seen if a less severe form of cardiac injury (such as ischemia-reperfusion) had been employed instead of permanent coronary artery ligation. On a different note, one may wonder whether there was any uptake of EVs by any organs after IV injections. Our previous studies have shown that there was significant hepatic uptake of EVs after IV administration; despite hepatic uptake after IV injection, there were no detectable differences in liver inflammation, fibrosis, or proliferation, which may be because this was not a liver disease model^[7,8,14]^. The main value of the negative hepatic findings is a grossly negative side effect profile to the liver tissue after IV HBMSC-EV injection, an increasingly common and non-invasive EV delivery method.

Secondly, how should studies showing improvement in cardiac function after systemic administration of EVs be interpreted when no significant cardiac uptake of EVs has ever been shown? One study systemically injected a porcine model of acute MI (via left circumflex artery ligation) with a 1,000 μg of mesenchymal stem cell-derived EV protein twice daily for 7 days following MI, and they achieved a clear reduction of infarct size (30%–40%) on cardiac MRI^[3]^. Cardiac MRI is a highly accurate modality and thus the improvements in cardiac function can be believed. However, one significant caveat to this study is the massive dose of the EVs used, which may not be practical given the vast quantity of cells, labor, supplies, and equipment needed to produce EVs - this would make the clinical use of EVs highly expensive and time-consuming for any benefit to be achieved. It is definitely possible that some EVs are taken up in the heart, as the current EV biodistribution detection methods such as fluorescent molecular tomography are not very sensitive, in that smaller IM EV doses are not detected^[7]^. Thus, if EVs are to be successfully transitioned to clinical use, more refined administration techniques will be needed or the cardiac-homing properties of EVs will need to be amplified.

In conclusion, a single-dose administration of neither IM nor IV injection of 2 × 10^9^ HBMSC-EV particles in a murine myocardial infarction model resulted in significantly improved cardiac function. This necessitates studies aimed at improving cardiac delivery by developing bioengineered EVs with cardiac or organ-specific delivery properties. The main limitation of this study is the lack of optimization of HBMSC-EV dose testing to see if increased doses or multiple doses could result in a post-MI cardiac benefit. However, the current study suggests that in order to treat cardiovascular disease efficiently in clinical settings - future studies are required to optimize EV dosage and administration routes, and/or bioengineer EVs to render cardiac-homing properties.

## Data Availability

Upon request from the first author, Xu CM.
